# High efficacy of huCD20-targeted AcTaferon in humanized patient derived xenograft models of aggressive B cell lymphoma

**DOI:** 10.1186/s40164-024-00524-4

**Published:** 2024-06-03

**Authors:** Willem Daneels, Alexander Van Parys, Leander Huyghe, Elke Rogge, Steffi De Rouck, Ruben Christiaen, Lennart Zabeau, Sylvie Taveirne, Jo Van Dorpe, Niko Kley, Anje Cauwels, Erik Depla, Jan Tavernier, Fritz Offner

**Affiliations:** 1 Department of Internal Medicine and Pediatrics, Ghent University, Ghent, Belgium; 2 Department of Hematology, Ghent University Hospital, C. Heymanslaan 10, 9000 Ghent, Belgium; 3 Cancer Research Institute Ghent (CRIG), Ghent University, Ghent, Belgium; 4 VIB-UGent Center for Medical Biotechnology, Ghent, Belgium; 5 Department of Biomolecular Medicine, Ghent University, Ghent, Belgium; 6 Orionis Biosciences BV, Ghent, Belgium; 7 Department of Diagnostic Sciences, Ghent University, Ghent, Belgium; 8 Department of Pathology, Ghent University Hospital, Ghent, Belgium

## Abstract

Type I interferon (IFN) is a potent antitumoral drug, with an important history in the treatment of hematologic malignancies. However, its pleiotropic nature leads to severe dose-limiting toxicities that blunt its therapeutic potential. To achieve selective targeting of specific immune or tumor cells, AcTakines (Activity-on-Target Cytokines), i.e., immunocytokines utilizing attenuated cytokines, and clinically optimized A-Kines™ were developed. In syngeneic murine models, the CD20-targeted murine IFNα2-based AcTaferons (AFNs) have demonstrated clear antitumoral effects, with excellent tolerability. The current study explores the antitumoral potential of the humanized huCD20-Fc-AFN in 5 different humanized patient derived xenograft (PDX) models of huCD20^+^ aggressive B non-Hodgkin lymphomas (B-NHLs). The huCD20-Fc-AFN consists of a huCD20-specific single-domain antibody (VHH) linked through a heterodimeric ‘knob-in-hole’ human IgG1 Fc molecule to an attenuated huIFNα2 sequence. An in vitro targeting efficacy of up to 1.000-fold could be obtained, without detectable in vivo toxicities, except for selective (on-target) and reversible B cell depletion. Treatment with huCD20-Fc-AFN significantly increased the median overall survival (mOS) in both non-humanized (mOS 31 to 45 days; HR = 0.26; p = 0.001), and humanized NSG/NOG mice (mOS 34 to 80 days; HR = 0.37; p < 0.0001). In humanized mice, there was a trend for increased survival when compared to equimolar rituximab (mOS 49 to 80 days; HR = 0.73; p = 0.09). The antitumoral effects of huCD20-Fc-AFN were partly due to direct effects of type I IFN on the tumor cells, but additional effects via the human immune system are essential to obtain long-term remissions. To conclude, huCD20-Fc-AFN could provide a novel therapeutic strategy for huCD20-expressing aggressive B-NHLs.

**Graphical Abstract:**

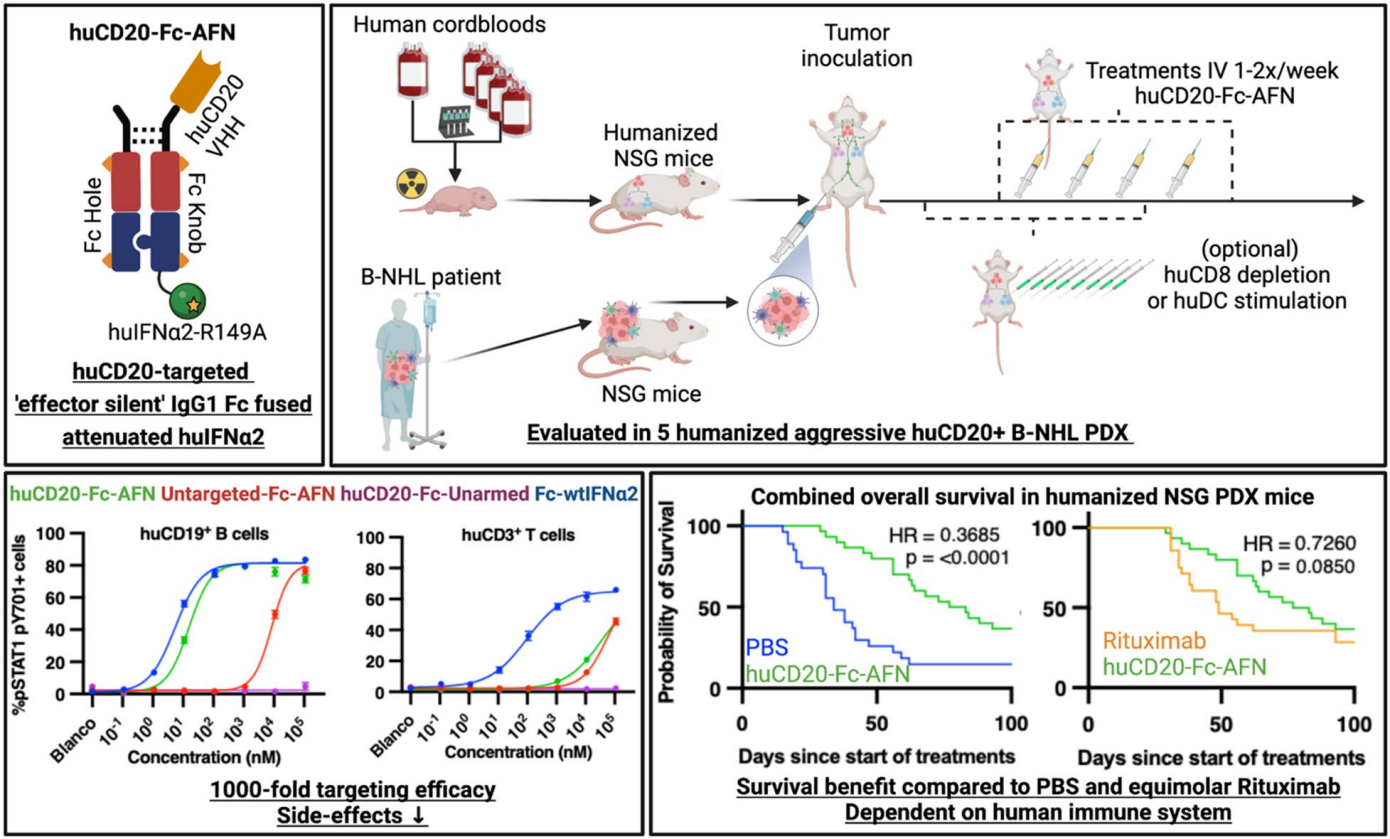

## Key points

- The A-Kine™-principle allows for up to 1.000-fold targeting efficacy, enabling reduced toxicities of IFNα2-based therapies.
- huCD20-Fc-AFN demonstrates significant antitumoral efficacy in 5 humanized PDX models of aggressive huCD20^+^ B-NHLs.

## Introduction

Mortality and morbidity related to malignant lymphomas remains a major global health issue, with a need for novel, effective, and less toxic treatments [1, 2]. Type I interferons (IFN) play a key driver role in the cancer-immunity cycle and are important for the success of many of the current antitumor therapies, including chemotherapy, radiotherapy, and immunotherapy [3]. There is an important history of IFN-based treatments in indolent B-NHLs, cutaneous T cell lymphoma, hairy cell leukemia, multiple myeloma, chronic myeloid leukemia and other myeloproliferative neoplasms [4]. Unfortunately, IFN-based cancer therapy experienced variable and unpredictable successes in the clinic, with dose-limiting toxicities due to their effect on nearly every cell type [5]. To reduce side effects, several strategies have been developed to specifically target cytokines to tumor cells or to the tumor niche, such as “immunocytokines”, composed of a cytokine, e.g. IFN, fused to a monoclonal antibody that recognizes a cell surface marker on the target cell [4, 6]. The use of attenuated cytokines has further improved their therapeutic index. Immunocytokines and their variants have been extensively discussed in several reviews [4, 7–11]. While many immunocytokines have demonstrated promising preclinical results, and some are being evaluated in ongoing clinical trials, none have thus far transitioned into routine clinical care.

Activity-on-Target Cytokines (AcTakines) and the clinically optimized A-Kines™, resemble classical immunocytokines, binding to their target cells using cell surface marker-specific ligands such as VHHs (antigen binding fragments of heavy chain only antibodies), but coupled to a mutant cytokine with strongly reduced binding affinity for their receptor complex, requiring up to 1.000-fold higher concentrations on untargeted cells to induce signaling [12–15]. In addition to the targeting moiety, the low-affinity mutant cytokines strongly reduce the interaction with cytokine receptors on untargeted cells while traveling through the body, limiting potential toxicities and sink effects [16]. Avidity effects at the targeted cell membrane lead to nearly full recovery of the cytokine signaling activity [13]. Previously, in vitro incubation with IFNα-based AcTakines, has demonstrated up to 1000-fold difference in EC50 for STAT1-pY701 induction, IFN-inducible firefly luciferase gene expression, inhibition of the cytopathic effect of the encephalomyocarditis (EMC) virus and cell proliferation of targeted versus untargeted cells. [13, 15] Finally, for a range of different mutant cytokines (IFN I & II, tumor necrosis factor (TNF), and interleukin-1 (IL-1), respectively referred to as AcTaferon I, II (AFN, AFN-II), AcTafactor (AFR) and AcTaLeukin I (ALN-I)) targeted directly to either tumor cells, specific immune cells or tumor neovasculature, the antitumoral potential has been demonstrated in several murine and human cancer models. [14, 15, 17–20]

The CD20 integral membrane protein is nearly exclusively expressed on B cells. Consequently, nearly all B-NHLs express CD20, be it at variable levels [21]. Most patients will receive rituximab or another CD20-targeted monoclonal antibody during their treatment [22]. In syngeneic mice, the efficacy of CD20-mAFN, a human IFNα2-Q124R mutant breaching the cross-species barrier and thus weakly active on murine cells, coupled either with a murine or human CD20 VHH, to respectively target the mCD20-expressing A20 murine B cell lymphoma cell line, and engineered hCD20-expressing murine B16 melanoma cell line, was previously reported with the following key findings: drastically reduced tumor growth only in targeted cell lines. This effect was completely lost in IFNAR– or Batf3–(Basic Leucine Zipper ATF-Like Transcription Factor 3) deficient mice. The antitumoral efficacy was depended on IFN signaling in conventional dendritic cells and the presence of, but not the IFN signaling in, CD8 + T lymphocytes. [17] The current study aims to further explore the antitumoral potential of huCD20-targeted huAFN in more advanced preclinical models, by mimicking the patient scenario as accurately as possible. Humanized patient derived tumor xenograft (PDX) models, which include a human patient-derived tumor, in combination with a human immune system (HIS), are a better approximation of the patient scenario [23, 24]. Indeed, compared to cancer cell lines, early passage PDXs more faithfully resemble the original tumors [25]. Additionally, drug response rates in PDX models have been shown to correlate with those observed in the clinic [26]. Because of the presence of human immune effector cells, humanized mouse models are increasingly being used to test and validate a broad range of immunotherapies [23, 27]. We here describe the antitumoral potential of huCD20-Fc-AFN compared to rituximab in 5 different humanized PDX models of huCD20^+^ aggressive B-NHLs.

## Methods

### Construction, production, and purification of human huCD20-Fc-AFN and control A-Kines

The huCD20 VHH and AFN (huIFNα2-R149A) sequences were cloned into in a heterodimeric, ‘knob-in-hole’ ‘effector-silent’ human IgG1 Fc context, resulting in the huCD20-Fc-AFN. Sequences were genetically linked via flexible GS-linkers. The resulting constructs were cloned in the pcDNA3.4 expression vector. To produce these ‘knob-in-hole’ Fc-constructs, a combination of knob and hole plasmids was transfected in ExpiCHO cells (ThermoFisher) according to the manufacturer’s instructions. One week after transfection, supernatant was collected, and cells removed by centrifugation. Recombinant proteins were purified based on protein A binding properties (Hitrap MabSelect SuRe column, GE Healthcare) and by subsequent size exclusion chromatography (Superdex 200 increase HiScale 16/40 column, GE Healthcare), both on an Äkta purifier (GE Healthcare). Concentrations were measured with a spectrophotometer (NanoDrop instrument, Thermo Scientific), purity estimated on SDS-PAGE and endotoxin-levels quantified on the EndoSafe Nexgen instrument (Charles River). For Untargeted-Fc-AFN, the huCD20 VHH was removed by standard mutagenesis techniques from the ‘knob’ construct. For huCD20-Fc-Unarmed, the sequence coding for the linker and warhead was deleted. Finally, for the positive controls, huCD20-Fc-wtIFNα2 and Untargeted-Fc-wtIFNα2, the *wild type* huIFNα2 sequence was cloned C-terminally of the ‘knob-in-hole’ Fc scaffold.

### Flowcytometric evaluation of PDX and human immune cells

Human immune cells were extracted from the spleen, bone marrow (femur), and PDX tumors by mechanical dissociation. Blood of humanized mice was harvested in K2-EDTA tubes. Human peripheral blood mononuclear cells (PBMCs) were isolated from healthy donor buffy coats. Murine and human Fc receptors were blocked using purified Rat Anti-Mouse CD16/CD32 and Human TruStain FcX™. Viability and surface stainings were performed using respectively LIVE/DEAD™ Fixable Aqua or Zombie NIR™ and combinations of huCD3-PacificBlue, huCD3-AlexaFluor700, huCD4-BV605, huCD5-AlexaFluor700, huCD8a-AlexaFluor700, huCD10-PE-Cy5, huCD19-APC, huCD19-BV605, huCD20-BV605, huCD20-PerCP, huCD33-PE, huCD33-FITC, huCD45-FITC, huCD45-APC, huCD45-BV570, muCD45-BV650, muCD45-PeVio615, huCD56-APC-Fire750, huCD70-APC, huKappa light chain-EFluor450, hu-Lambda light chain-PE, huPD-L1-Pe-Cy7, huPD-1-APC-Cy7 (Biolegend/Invitrogen/Miltenyi Biotec). After cell surface staining, cells were stimulated for 15 min, fixed and permeabilized using BD Phosflow Fix I and Perm III, and subsequently incubated overnight with anti-human/mouse STAT1 pY701-PE (Miltenyi Biotec). Samples were measured on the MACSQuant® Analyzer 16 (Miltenyi Biotec) and analyzed in FlowLogic 8.6 (Inivai Technologies).

### In vitro cytotoxicity assays

CellTiter-Glo® luminescent Cell Viability Assay (Promega) was performed as per manufacturing guidance. Cells were incubated for 24–96 h at 37 °C in RPMI 1640 + 10% FBS. Given the poor in vitro proliferation of PDX cells, we started with a 20.000 cells/well to measure in vitro survival rather than proliferation. Measurements were performed on EnSight™ Multimode Plate Reader (PerkinElmer) and analyzed using the Kaleido™ Data Acquisition software (PerkinElmer).

### In vivo experiments

A detailed description of the different experimental setups can be found in Table 1. Compounds were administered IV by tail vein injections, in a 100–200 μL volume, using individual, sterile, 29G needles. HuCD20-Fc-AFN was dosed at 40 μg once or twice per week, depending on the experiment (Table 1). Clinical grade rituximab (Mabthera, Roche) was equimolarly dosed (63 μg). Doxorubicin (Adriblastina, Pfizer) was dosed at 25 μg/wk (± 1mg/kg). Treatments were started at either a fixed timepoint or fixed tumor volume of 75 mm^3^. For cDC1 stimulation, 30 μg huFlt3L was administered IP once daily between D-2 and + 2, and between D + 4 and + 8 (8 administrations). For huCD8 depletion, InVivoMAb™ anti-human CD8α (#BE0004-2, clone OKT8, Bio X Cell) was administered 2x/week IP for 4 weeks (200 μg W1; 100 μg W2–3–4), starting D-2. The evaluation of the LY13005 model was performed at the research facilities of the clinical research organization Experimental Pharmacology & Oncology Berlin-Buch GmbH (EPO), with treatments blinded to the local researchers to guarantee an external, independent evaluation.

**Table 1:** In vivo experimental setup

PDX	Figure reference	Mice per group	Tumor cells injected	Start of treatments	huCD20-Fc-AFN treatment	Other treatments	huCD8 depletion	huFlt3L administration
LY3005	Figures 9B, 10, 13B	4–5	Fragment 3 × 3 mm	Tumor volume > 75 mm^3^	40 μg 2x/wk × 8	PBS, Rituximab^a^, Untargeted-Fc-AFN^a^, huCD20-Fc-Unarmed^a^	No	Yes^c^
LYM011	Figures 9B, 10, 13B	5–7	10 × 10^6^	Tumor volume > 75 mm^3^	40 μg 2x/wk × 8	PBS, Rituximab^a^, Untargeted-Fc-AFN^a^, huCD20-Fc-Unarmed^a^	No	Yes^c^
LYM024	Figures 9A, 13A	6	0,6 × 10^6^	D + 11	40 μg 1x/wk × 4	PBS, Rituximab^a^	No	No
LYM024	Figure 12B	6–7	1–2 × 10^6^	D + 13	40 μg 1x/wk × 4	PBS	Yes^b^	No
LYM034	Figures 9A, 13A	6–7	2 × 10^6^	D + 11	40 μg 1x/wk × 4	PBS, Rituximab^a^	No	No
LYM034	Figure 10	6–7	1 × 10^6^	Tumor volume > 75 mm^3^	40 μg 2x/wk × 8	PBS, Untargeted-Fc-AFN^a^	No	Yes^c^
Proxe_MCL	Figures 9A, 13A	6	2 × 10^6^	D + 11	40 μg 1x/wk × 4	PBS, Rituximab^a^	No	No
Proxe_MCL	Figures 11A, B	7–9	1.25 × 10^6^	Tumor volume > 75 mm^3^	40 μg 2x/wk × 8	PBS	No	Yes^d^

Overview of the exact in vivo experimental setup for each figure in this manuscript regarding PDX model, number of mice per group, number of tumor cells used for tumor inoculation, dose of huCD20-Fc-AFN, start of treatment, other treatments in same experiment and the use of huCD8 depletion and or huFlt3L administration

D: Day; PBS: Phosphate-buffered saline; AFN: AcTaferon

^a^Equimolar to huCD20-Fc-AFN

^b^huCD8 depletion with OKT8 2x/week IP (Week 1: 2 × 200 μg – Week 2–3-4: 100 μg) – start D-2

^c^30 μg daily IP injection for 2 × 4 days from day-2 to day 10

^d^30/3/0μg daily IP injection for 2 × 4 days from day-2 to day 10

### Animal housing and humane endpoints

Immunodeficient NOD.Cg-*Prkdc*^*scid*^*Il2rg*^*tm1Wjl*^/SzJ (NSG) mice, originally purchased from The Jackson Laboratory, were bred in-house, housed in individually ventilated cages, grown in pathogen-free conditions, without antibiotic treatments, in a temperature-controlled environment with a 12/12-h light/dark cycle with food and water supply ad libitum. Mice were checked daily. Tumor volume and animal weight were measured 2-3x/week. Mice were sacrificed in case of tumor volume > 1500 mm^3^ (L*W^2^/2), overt organomegaly, weight loss > 20% or development of significant xenogeneic graft-versus-host-disease (xGVHD) such as > 30% hair loss, hunching posture, decreased mobility or overt anemia. [28, 29]

### Humanization process

**Fig. 1:**
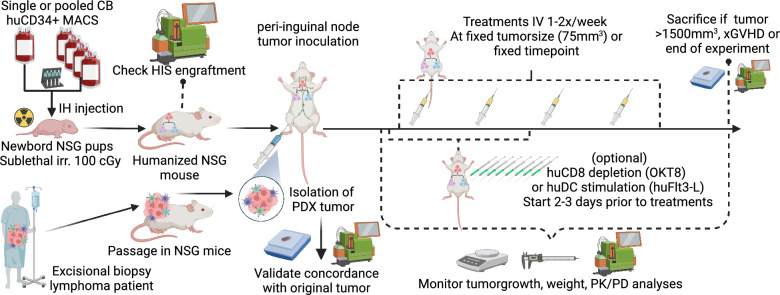
Schematic overview of humanized PDX generation and experimental setup. Newborn NSG mice are humanized using CD34^+^ MACS selected HSC derived from umbilical cord bloods, engraftment was checked by flow cytometry. Humanized mice were inoculated with early-passage PDX tumors freshly isolated from non-humanized NSG mice. Intravenous treatments were started at fixed timepoints or tumor size, optionally preceded by intraperitoneal huCD8 depletion or huDC stimulation. See text for further details, PDX characteristics can be found in Table 2 and Table 1 for specific experimental conditions. *MACS*: *Magnetic-activated cell sorting; NSG*: *NOD scid gamma; IH*: *intrahepatic; cGy*: *centigray; IV*: *intravenous; xGVHD*: *xenogeneic graft-versus-host-disease; huDC*: *human dendritic cells; PK/PD*: *pharmacokinetics/dynamics; CB*: *Cord blood*

A graphical overview of our humanization process is presented in Fig. 1 and was previously described in detail [17]. Briefly, newborn NSG pups were irradiated with 100 cGy and intrahepatically injected with 1 × 10^5^ CD34^+^ hematopoietic stem cells (HSCs) isolated by Magnetic-Activated Cell Sorting (MACS) with the CD34 MicroBead Kit (Miltenyi Biotec) from Lymphoprep™ density gradient centrifugated human umbilical cord blood, obtained from the Cord blood Bank and Hematopoietic Stem cell Bank of the Ghent University Hospital, Belgium. Engraftment was evaluated at week 12 by flow cytometry. Only mice with a clear (> 5%) circulating huCD45^+^ population were used. For the LY13005 model, humanization was performed at EPO by intravenous injection into three-week old, irradiated, NOD.Cg-*Prkdc*^scid^*Il2rg*^tm1sug^/JicTac (NOG) mice. While engraftment of human immune cells in NSG and NOG mice is comparable, engraftment is generally more efficient in newborn mice compared to adult mice. [23, 24, 30, 31]

### PDX model generation

**Fig. 2:**
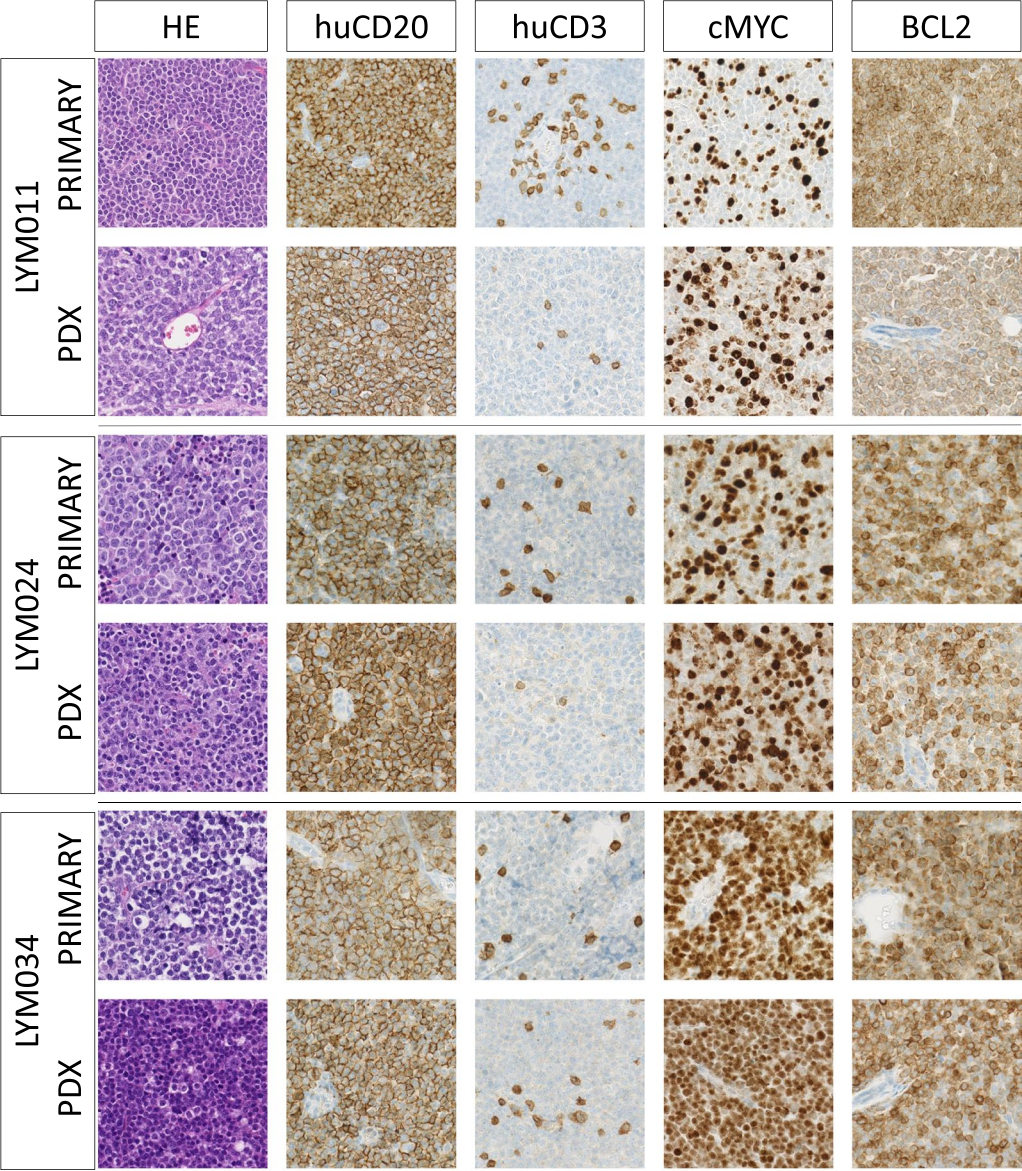
Concordance of IHC findings between primary patient tumor and PDX. Comparison of immunohistochemistry (IHC) findings between the original tumor biopsy in the patient and early passage PDX tumor in humanized mice. The expression patterns of huCD20, cMYC and BLC2 matched their original phenotype. In humanized mice we observed influx of huCD3^+^ cells. Staining was performed on formalin fixed paraffin embedded (FFPE) tissues at the Laboratory of Pathological Anatomy at the Ghent University Hospital. Images captured on NanoZoomer-RS at 40 × magnification and processed in NDP.view2. PDX tumors were derived from humanized mice at passage P3/P2/P1 in LYM011/LYM024/LYM034, respectively. PDX characteristics can be found in Table 2

**Fig. 3:**
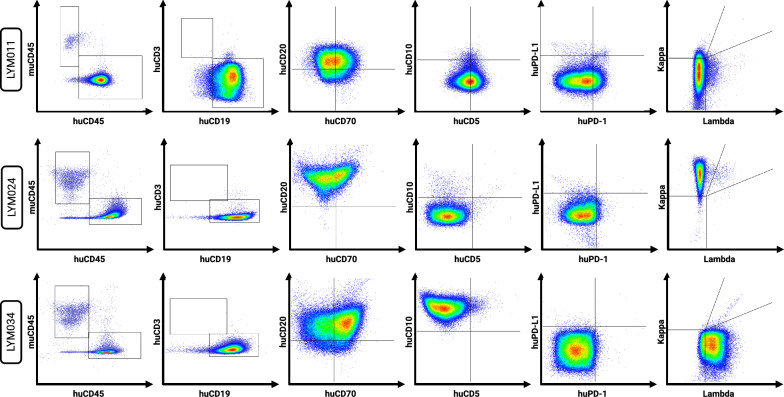
Flowcytometric profile of the lymphoma PDX models. Our in-house developed PDX models (LYM011, LYM024 and LYM034) were evaluated for different cell surface markers (mu/huCD45, huCD3, huCD19, huCD20, huCD70, huCD10, huCD5, huPD-L1, huPD-1, surface Kappa light chains, surface Lambda light chains). Analyses performed in low-passage PDX tumors derived from non-humanized NSG mice. No permeabilization-step was performed. All plots are gated on the live huCD45^+^ cell proportion, with the exception of the far-left plot for each model (being the human versus murine CD45 gating itself)

Figure 1 and Table 2 provide an overview of the evaluated models and workflow. The Proxe_MCL model was derived from DFBL-98848-V3-mCLP, purchased from the Public Repository of Xenografts (PRoXe) at the Leukemia and Lymphoma Xenograft (LLX) core laboratory at Dana-Farber Cancer Institute (DFCI) [32]. The LY13005 model was developed by EPO [33]. We independently developed the PDX models LYM011, LYM024, LYM034 from fresh residual tissue from diagnostic excision biopsies from lymphoma patients treated at the Ghent University Hospital. Four-week-old NSG mice were irradiated with 200 cGy prior to tumor inoculation. Tumors were serially transplanted into unirradiated NSG mice and/or cryopreserved. All experiments were performed with PDX tumors with $\le$ 3 serial passages. Concordance with the original tumor phenotype was evaluated by flow cytometry and immunohistochemistry (IHC) for CD20/CD3/BLC2/BCL6/cMYC expression. Figure 2 illustrates comparative IHC findings and Fig. 3 the flowcytometric profile for the in-house developed PDX models.

**Table 2:** Characteristics of the PDX models used: subtype, exposure to prior treatments before biopsy, and origin

PDX Name	Subtype	Prior therapy	Origin
LYM011	Richter transformation of CLL (DLBCL)	Relapse/transformation < 1 year after 6 cycles of rituximab + bendamustine for CLL	Ghent University Hospital, Belgium
LYM024	Diffuse large B-cell lymphoma, non-GC subtype, BCL2 and cMYC double expressor, no *BCL2* or *cMYC* rearrangement, CISH EBV (EBER) negative	Partial response to 6 cycles of R-CHOP + 4 × IT MTX + 2 × rituximab monotherapy	Ghent University Hospital, Belgium
LYM034	High-grade-B-cell-lymphoma, NOS (*MYC* rearrangement), strong BCL2 expression	Relapse < 1 year after 6 cycles of R-CHOEP + 4 × IT MTX + 2 × rituximab monotherapy	Ghent University Hospital, Belgium
Proxe_MCL	Mantle Cell lymphoma	Rituximab + bendamustine	Public Repository of PDX (PRoXe), Leukemia/Lymphoma Xenograft (LLX) Core Laboratory at Dana-Farber Cancer Institute, USA
LY3005	Diffuse large B-cell lymphoma–ABC subtype	Untreated	Experimental Pharmacology and Oncology (EPO), Berlin, Germany

*DLBCL*: *diffuse large B cell lymphoma; BCL2*: *B cell lymphoma 2; ABC*: *Activated B cell; NOS*: *not otherwise specified; CLL*: *Chronic lymphocytic leukemia; IT*: *Intrathecal; MTX*: *Methotrexate; R-CHOP*: *rituximab* + *cyclophosphamide* + *hydroxodaunorubicin* + *vincristin* + *prednisolone; R-CHOEP*: *rituximab* + *cyclophosphamide* + *hydroxodaunorubicin* + *etoposide* + *vincristin* + *prednisolone; CISH*: *Chromogenic *in situ* hybridization; EBV*: *Epstein–Barr virus; EBER*: *Epstein–Barr virus–encoded small RNAs*

### Statistics

Analyses and graphs were produced in Prism (Graph Pad, version 10). Survival analyses were performed using Kaplan Meier analyses, Gehan-Breslow-Wilcoxon test, and Mantel–Haenszel Hazard Ratios. For combining multiple PDX models, all individual datapoints were added to the Kaplan Meier analysis to balance their relative contribution. Differences in tumor volumes or immune cells were compared using a Welch and Brown-Forsythe ANOVA followed by Dunnett’s T3 multiple comparisons test with significance levels set at 0.05. Standard curves and half maximal effective concentration (EC50) were calculated using nonlinear regression with variable slope. Error bars represent the standard error of the mean (SEM). Mice sacrificed because of tumor volumes $\ge$ 1500 mm^3^, retained this value until the end of the experiment to avoid incorrect improvement of grouped tumor volumes due to dropouts. Linear point-to-point interpolation between measurements was performed to correct for missing values at certain timepoints when treatments were started individually. Graphs with tumor volumes show either measured of interpolated datapoints at fixed 3-day intervals. Survival curves consist of only actual datapoints.

## Results

### Construct design for selective targeting of huCD20^+^ cells

**Fig. 4:**
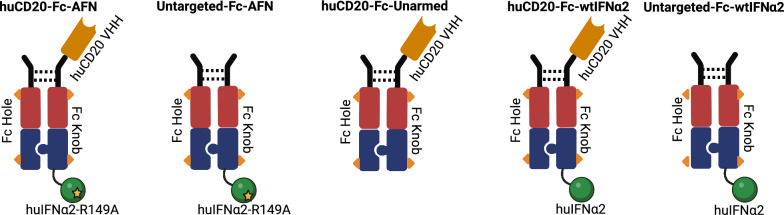
Schematic representation of huCD20-Fc-AFN, Untargeted-Fc-AFN, huCD20-Fc-Unarmed, huCD20-Fc-wtIFNα2 and Untargeted-Fc-wtIFNα2. Overview of the different constructs. Solid lines represent GS-linkers, dashed lines disulphide bridges, triangles the Fc “effector silencing” modifications, and the star the A-Kine R149A mutation. See also text for more details

Figure 4 illustrates the design of huCD20-Fc-AFN, consisting of a huCD20-targeted VHH, linked through a heterodimeric ‘knob-in-hole’ IgG1 Fc molecule to the mutated huIFNα2 sequence. The first subunit consists of the huCD20 VHH sequence, fused to a human IgG1 Fc sequence containing effector knock-out mutations plus ‘knob’ modifications, and the huIFNα2 sequence with the R149A mutation [34]. The second subunit consists of a human IgG1 Fc sequence with effector knock-out mutations and ‘hole’ modifications. The ‘knob-in-hole’ principle promotes the heterodimer formation, by manipulating key complementary amino acid residues on the Fc sequence [35]. Effector knock-out mutations on the human IgG1 Fc were induced to impede interaction with innate immune cells through the Fcγ receptors (FcγRs) and complement component 1q (C1q). These mutations impede the induction of antibody-dependent cellular cytotoxicity (ADCC), antibody dependent cellular phagocytosis (ADCP) and complement-dependent cytotoxicity (CDC) by the Fc fragment. The use of an ‘effector-silent’ Fc variant limits the potential for infusion reactions and unwanted activation of untargeted effector cells [35–38]. Importantly, the recognition motifs for the neonatal Fc receptor (FcRn), necessary for half-life extension by avoiding lysosomal degradation through FcRn-mediated recycling, remained unchanged [35, 36, 38]. The R149A mutation in the huIFNα2 sequence significantly reduces the affinity for the IFNAR-2 subunit of type I IFN receptor [13, 39, 40]. However, avidity effects can restore proper signaling [13, 17]. Fig. 4 also depicts the relevant controls used: Untargeted-Fc-AFN (no huCD20 VHH), huCD20-Fc-Unarmed (no AFN warhead), huCD20-Fc-wtIFNα2 and Fc-wtIFNα2 (*wildtype* huIFNα2 instead of AFN with or withing the huCD20 VHH). Further details can be found in the methods section above.

**Fig. 5:**
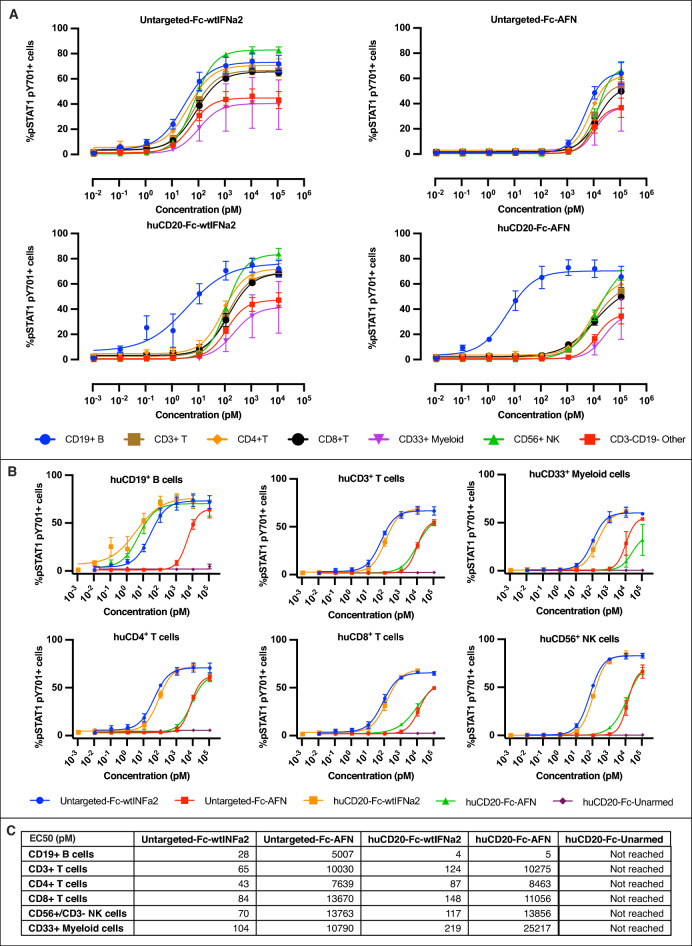
High in vitro targeting efficacy of huCD20-Fc-AFN by pSTAT1 induction in PBCM's. **A** Concentration dependent pSTAT1 pY701 induction in human immune cell subtypes by flowcytometry, upon 15 min in vitro stimulation of PBMCs with the different constructs. **B** Comparison of pSTAT1 pY701 induction within the different human immune cell subtypes of the different construct by flowcytometry, upon 15 min in vitro stimulation of PBMCs.**C** Half maximal effective concentration (EC50) in pM for each construct, on different human immune cells. No reliable EC50s for huCD20-Fc-Unarmed could be calculated and values are thus not displayed. *EC50*: *Half maximal effective concentration*

**Fig. 6:**
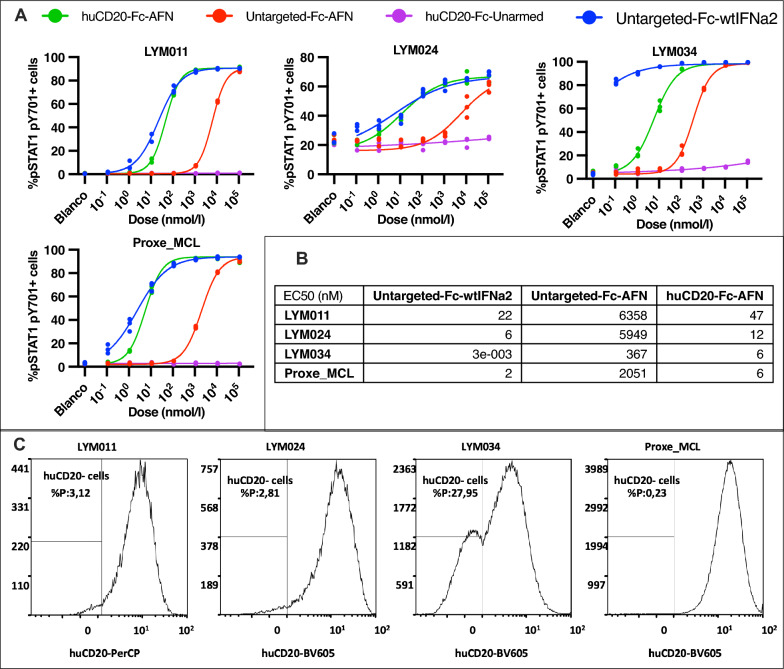
HuCD20-Fc-AFN selectively induces pSTAT1 in the PDX models used. **A** %pSTAT1 pY701 positive PDX tumor cells by flowcytometry, upon 15 min in vitro stimulation with the different constructs. Except for LYM034, the effect of huCD20-Fc-AFN was comparable with Fc-wtIFNα2. Untargeted-Fc-AFN induced pSTAT1 only at up-to 3.000-fold higher concentrations. No effect on pSTAT1 was seen with huCD20-Fc-Unarmed. **B** Half maximal effective concentration (EC50) in pM for each construct on different PDX models, using the maximal effect of Untargeted-Fc-wtIFNα2 as a reference. No reliable EC50 for huCD20-Fc-Unarmed could be calculated. **C** Histogram of huCD20 expression on flowcytometry of live huCD45^+^ PDX cells. A significant huCD20 negative subpopulation is present in LYM034 compared to the other PDX models. *EC50*: *Half maximal effective concentration; %P* = *% of parent population (live huCD45*^+^ *cells); BV605*: *Brilliant violet 605; PerCP*: *Peridinin-Chlorophyll-Protein*

Among others, signal transducer and activator of transcription 1 (STAT1) is recruited to the activated type I IFN receptor and phosphorylated by the associated kinases [41, 42]. Fig. 5A, B demonstrate the effects on pSTAT1 pY701 in PBMCs after in vitro stimulation. Due to competitive binding of the huCD20 VHH with flow cytometry antibodies on the huCD20-epitope, huCD19 was chosen as a surrogate marker for huCD20^+^ PBMCs and PDX cells. In on-target huCD19^+^ B cells, huCD20-Fc-AFN induced pSTAT1 comparable to Fc-wtIFNα2 and huCD20-Fc-wtIFNα2. The effect of huCD20-Fc-AFN on untargeted T/NK/Myeloid cells was comparable to Untargeted-Fc-AFN (Fig. 5B). This in contrast to huCD20-Fc-wtIFNα2, where pSTAT1 induction in untargeted cells was comparable with Untargeted-Fc-wtIFNα2. The latter indicates that coupled to the highly reactive wtIFNα2-warhead, the huCD20-VHH itself provides limited targeting efficacy. Finally, no increase in pSTAT1-pY701 was seen with huCD20-Fc-Unarmed. Together, these findings revealed in vitro targeting efficacies up to 1.000-fold, largely due to the attenuated AFN-warhead, with an EC50 in the picomolar range on target cells for huCD20-Fc-AFN (Fig. 5C). Additionally, the pSTAT1 profile upon in vitro stimulation was comparable for huCD20-Fc-AFN or Untargeted-Fc-wtIFNα2 in all evaluated PDX models (Fig. 6A), except for LYM034, which harbored a significant huCD20^−^ subpopulation (Fig. 6B).

### IFN/AFN signaling does not induce direct cytotoxic effects in lymphoma PDX cells

**Fig. 7:**
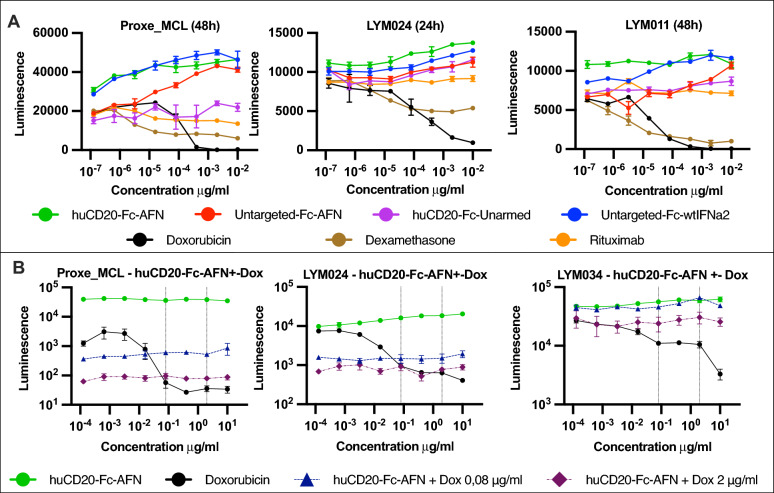
In vitro survival advantage of huCD20-Fc-AFN on lymphoma PDX cells by CellTiter-Glo assay. ** A** Luminescence from CellTiter-Glo assay, measuring cellular ATP after 24–48 h incubation with different compounds. Luminescence positively correlates with AFN/IFN concentration in 3 different PDX models, suggesting an in vitro survival benefit. Matching their lymphotoxic properties, luminescence negatively correlates with dexamethasone and doxorubicin concentrations. Rituximab and huCD20-Fc-Unarmed show little effect. **B** Luminescence from CellTiter-Glo assay after 48h incubation of PDX cells. Concentrations indicated are for huCD20-Fc-AFN in the green, blue, and purple curves, and doxorubicin for the black curve. Additionally, a fixed dose of doxorubicin (0,08 μg/ml or 2 μg/ml) was added to the dotted blue and purple curves, respectively. The dotted vertical lines intersect at these concentrations, illustrating the corresponding effect of doxorubicin alone. Addition of huCD20-Fc-AFN increases luminescence and thus (partially) overcomes the cytotoxic effect of doxorubicin in vitro on PDX cells. *dox*: *doxorubicin; AFN*: *AcTaferon; IFN*: *Interferon*

We investigated the direct in vitro effects on PDX tumor cell survival upon 24–48 h incubation with huCD20-Fc-AFN or other treatments. Figure 7A illustrates a CellTiter-Glo assay, which measures cellular ATP and thus quantifies metabolically active cells. As expected, known lymphotoxic drugs, such as dexamethasone and doxorubicin, induced a concentration-dependent decrease in luminescence. However, a paradoxical increase in luminescence was observed with increasing huAFN/IFNα2 concentrations. The effect of huCD20-Fc-AFN was comparable to Fc-wtIFNα2. Untargeted-Fc-AFN only produced a similar effect at the highest concentrations, consistent with the targeting efficacies demonstrated above with pSTAT1. No effects of rituximab, nor of huCD20-Fc-Unarmed, were observed. Hence, in the absence of effector cells, in vitro huCD20 targeting alone was not directly cytotoxic in these PDX models. Surprisingly, huCD20-Fc-AFN could partially abrogate the cytotoxic effect of doxorubicin (Fig. 7B).

### PK/PD profile of huCD20-Fc-AFN is dependent on humanization

**Fig. 8:**
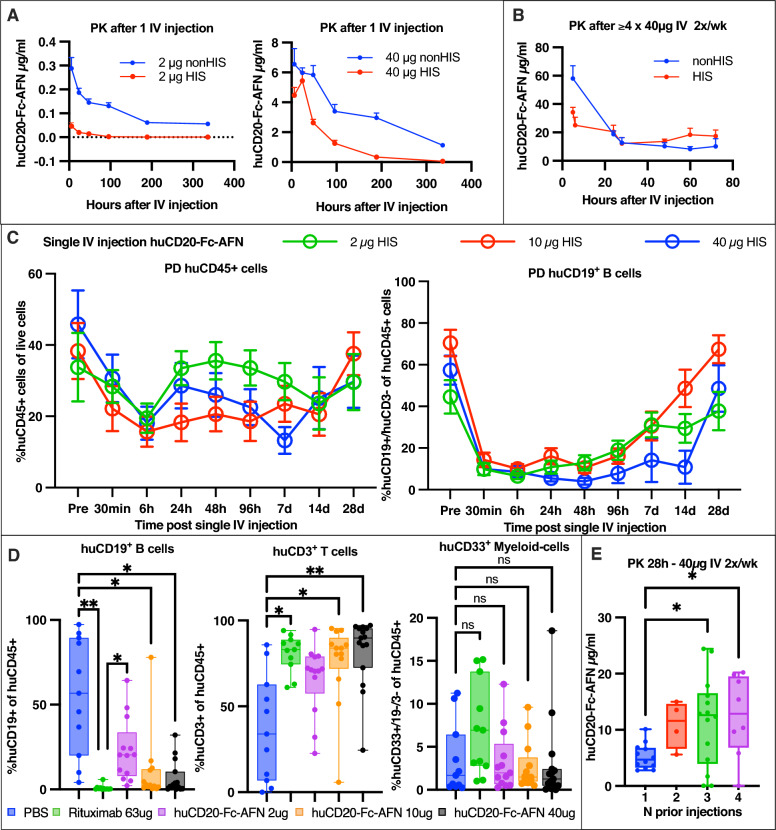
PK/PD profile of huCD20-Fc-AFN in HIS/nonHIS NSG mice. **A** PK profile of a single IV injection of huCD20-Fc-AFN in HIS/nonHIS NSG mice. A clear sink-effect due to the HIS compartment is observed, which is more pronounced in the lowest dose. **B** PK profile from last IV injection of 40 μg huCD20-Fc-AFN in HIS/nonHIS mice who have received ≥ 4 IV injections 2x/wk. Compared to a single dose, increased plasma concentrations, which are less HIS dependent are observed. **C** Effect on huCD45 + human cells and huCD19 + B cells in the blood (using huCD19 as surrogate marker for huCD20) after a single IV injection, demonstrating the rapid, selective, and reversible B cell depletion. This lasts up to 14 days in a dose-dependent manner. **D** demonstrates the PD effects 72 h after second administration of 40 μg huCD20-Fc-AFN or equimolar rituximab 2x/week. Each dot represents one humanized NSG mouse. Analyses by flow cytometry on live cells after RBC-lysis. We can observe a selective B cell depletion with relative increase in absolute T cells without any effects on myeloid cells for both drugs. **E** demonstrates the accumulation of huCD20-Fc-AFN with repeated 40 μg 2x/week injections. PK: pharmacokinetics; PD: pharmacodynamics; IV: intravenous; HIS: Humanized immune system; ns = p > 0.05; *p ≤ 0.05; **p ≤ 0.01; AFN: AcTaferon

As cytokines are generally short-lived, previously published AFNs targeting murine cells in syngeneic models had to be administered perilesional to avoid daily IV injections. Fc-fusion, as described above, significantly induces half-life extension by FcRn-mediated recycling and reduced renal clearance, allowing for once-weekly IV administration [43, 44]. Fig. 8 illustrates the PK/PD profile after single or repeated IV injections. A profoundly different PK profile is observed, depending on the administered dose, number of prior injections and humanization status. In non-humanized mice, plasma concentrations well over the EC50 for targeted cells were still observed 14 days after a single IV injection. However, in humanized mice, plasma concentrations decreased much more rapidly, and this effect was dose dependent (Fig. 8A). However, the PK profile after repeated higher dose injections was similar in HIS and nonHIS mice (Fig. 8B). Additionally, huCD20-Fc-AFN accumulated when administered at 40 μg 2x/week (Fig. 8E). Together, these findings imply an important on-target off-tumor sink effect due the human B cell compartment in humanized mice (further detailed below).

The PD profile in Fig. 8C, D illustrate the in vivo selectivity of huCD20-Fc-AFN. After a single injection in humanized NSG mice, we observed a rapid, selective, and reversible decline of on-target human B cells from the peripheral blood, which resolved in a dose-dependent manner after 7–28 days (Fig. 8C). No off-target effects could be observed, besides the initial decrease of total huCD45^+^ cells, with a relative increase in T cells due to depletion of the B cells (Fig. 8C, D). The latter was not observed with non-B cell-targeted variants. Additionally, Fig. 8D further illustrates the selective, near complete depletion of B cells, 72 h after the second administration of huCD20-Fc-AFN or rituximab.

### Strong antitumoral effect in different PDX models

**Fig. 9:**
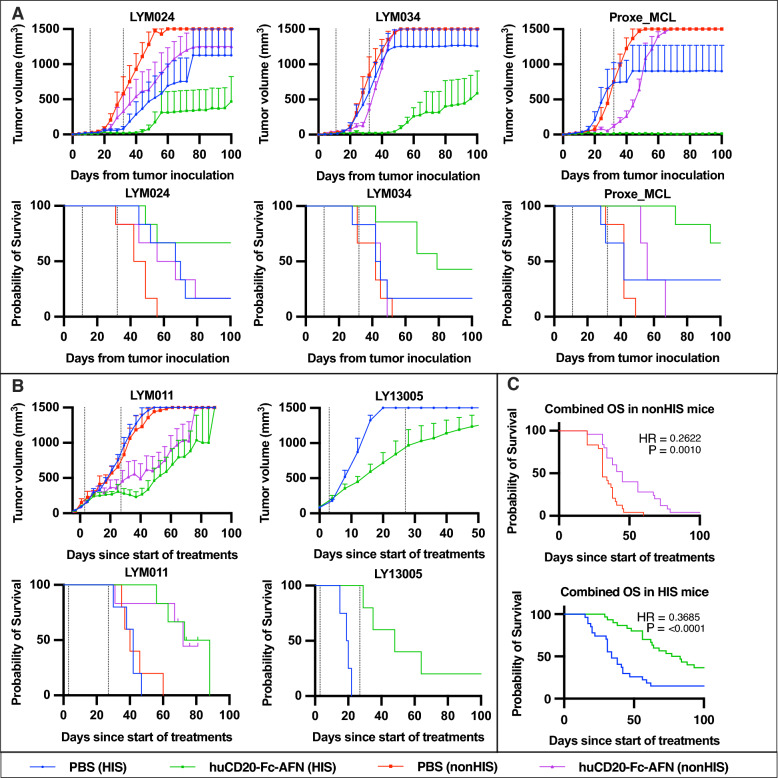
Antitumoral effect of huCD20-Fc-AFN in 5 different humanized PDX lymphoma models. **A** Tumor volumes and survival from tumor inoculation. Administration of huCD20-Fc-AFN 40 μg 1x/week for 4 weeks from D11. HIS and nonHIS NSG (n = 7/group). Dotted lines indicate the first and last treatment. **B** Tumor volumes and survival from start of treatment. Administration of huCD20-Fc-AFN 40 μg 2x/week for 4 weeks, started on an individual basis when tumor volume reached > 75 mm^3^. Pretreatment with IP 30 μg huFlt3L 2 × 4 days, starting D-2. For LY13005 only HIS NOG mice used (n = 4–5/group), for LYM011 HIS and nonHIS NSG mice (n = 5–7/group). Dotted lines indicate the first and last treatment. **C** Kaplan Meier survival curve of combined data from experiments in panel A + B. Survival from start of treatment for n = 24 vs 25 and n = 27 vs 30 in PBS vs huCD20-Fc-AFN in nonHIS and HIS mice respectively. A significant difference in overall survival is seen in both nonHIS and HIS mice. *OS*: *overall survival; HR*: *Hazard ratio; HIS*: *human immune system; PBS*: *Phosphate-buffered saline; AFN*: *AcTaferon*

**Fig. 10:**
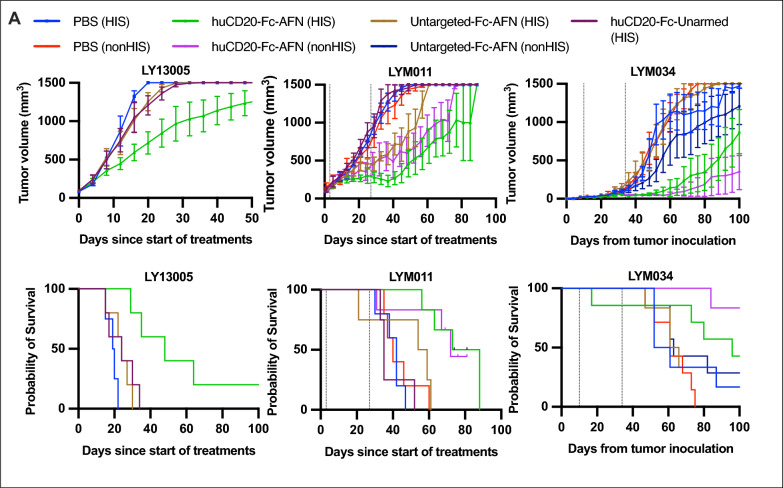
Untargeted-Fc-AFN or huCD20-Fc-Unarmed do not induce significant antitumoral effects. Tumor volumes and survival from start of treatment (LY13005 and LYM011) or tumor inoculation (LYM034). Administration of huCD20-Fc-AFN 40 μg or equimolar Untargeted-Fc-AFN or huCD20-Fc-Unarmed 2x/week for 4 weeks, started on an individual basis when tumor volume reached > 75 mm^3^. Pretreatment with IP 30 μg huFlt3L 2 × 4 days, starting D-2. For LY13005 only HIS NOG mice used (n = 4–5/group), for LYM011 and LYM034 HIS and nonHIS NSG mice (n = 5–7/group). Dotted lines indicate the first and last treatment. Untargeted-Fc-AFN and huCD20-Fc-Unarmed did not induce similar antitumoral responses compared to huCD20-Fc-AFN. PDX characteristics can be found in Table 2

Figure 9 demonstrates the antitumoral potential of 40 μg huCD20-Fc-AFN IV 1 or 2x/week (respectively, Fig. 9A, B) for 4 weeks in HIS and nonHIS mice in 5 separate PDX models. All models were derived from aggressive B-NHLs, of which 80% priorly exposed to rituximab (Table 2). Figure 1 provides a schematic overview of the methodology, and Table 1 the exact experimental setup for each experiment. Tumor growth was generally suppressed during treatment. In nonHIS mice, tumor growth resumed upon cessation of treatment. However, in HIS mice, several tumors did not relapse by day 100, suggesting a cure. No loss of huCD20 expression was observed in tumors that progressed or relapsed after huCD20-Fc-AFN treatment. The antitumoral effect was present in both strains of humanized mice (NOG in LY13005 and NSG in all others). While no antitumoral effect was observed with huCD20-Fc-Unarmed, a limited effect was observed from the untargeted residual activity of Untargeted-Fc-AFN (Fig. 10). To more reliably quantify the antitumoral effect, we combined the survival data from the 5 models (Fig. 9C). Administration of huCD20-Fc-AFN significantly increased the median overall survival (mOS) from start of treatment in both nonHIS (mOS 31 to 45 days; HR = 0.26; p = 0.001), and HIS mice (mOS 34 to 80 days; HR = 0.37; p < 0.0001).

### Analysis of the role of human dendritic and CD8^+^ T cells to the antitumoral effects of huCD20-Fc-AFN

**Fig. 11:**
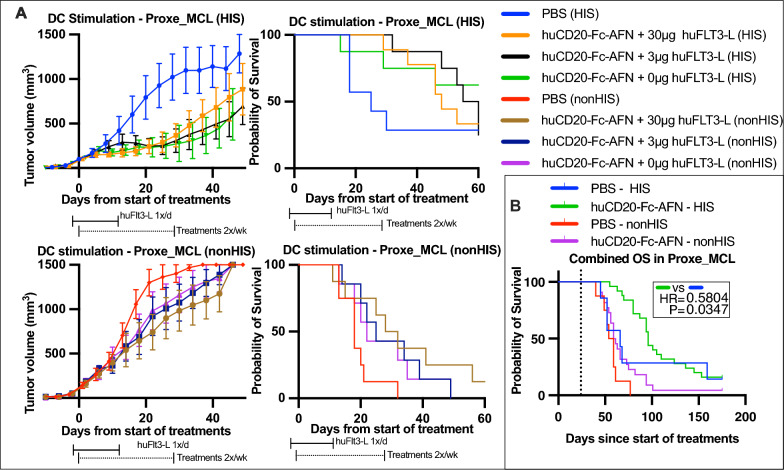
The impact of cDC1 stimulation by huFlt3-L administration on the efficacy of huCD20-Fc-AFN. **A** Tumor volumes and survival from start of treatment. Administration of huCD20-Fc-AFN 40 μg 2x/week for 4 weeks, started on an individual basis when tumor volume reached > 75 mm^3^. DC Stimulation by pretreatment with 30/3/0 μg huFlt3L 2 × 4 days IP, starting D-2. No significant differences were noted between huFlt3L doses. Suppression of tumor growth, and thus survival, was more pronounced in HIS mice. HIS and nonHIS NSG (n = 7–9/group). **B** Kaplan Meier survival curve of combined data from experiment in panel A. Survival from start of treatment for n = 8 vs 22 and n = 7 vs 25 in PBS vs huCD20-Fc-AFN in nonHIS and HIS mice, respectively. A significant difference in overall survival was seen in HIS mice only

Previously, the antitumoral efficacy of muCD20-AFN in syngeneic mouse models required the presence, and proper IFN I signaling of murine cDC1s, as its effect was lost in cDC1-deficient Batf3^−/−^ mice and CD11c-IFNAR^−/−^ mice [15]. Administration of exogenous huFlt3L (human Fms Related Receptor Tyrosine Kinase 3 Ligand) increases the number, and activation status of cross-presenting cDC1s [45–47]. The latter are critical to elicit strong CD8^+^ T cell-mediated antitumoral effects [48]. Additionally, exogenous huFlt3L administration seemed necessary for the antitumoral effect of cDC1-directed AFN-constructs [49, 50]. To determine the effect of huFlt3L on huCD20-Fc-AFN efficacy, we compared the addition of intraperitoneal (IP) administration of 30 μg, 3 μg or 0 μg huFlt3L for 8 days, starting 2 days prior to huCD20-Fc-AFN treatments in the Proxe_MCL model (Fig. 11A). Unexpectedly, we observed that the antitumoral effect of huCD20-Fc-AFN was not influenced by huFlt3L treatments. However, a clear difference in tumor growth was noted depending on the presence of the human immune system. In the PBS groups, humanization induced a delayed tumor growth, albeit not statistically significant. In the huCD20-Fc-AFN groups, survival was only significantly different compared to PBS in HIS mice (mOS 66 to 96 days; HR = 0.58; p = 0.03) (Fig. 11B). These findings indicate a substantial contribution of the human immune system to huCD20-Fc-AFN efficacy in humanized NSG mice, independent of exogenous huFlt3L administration.

**Fig. 12:**
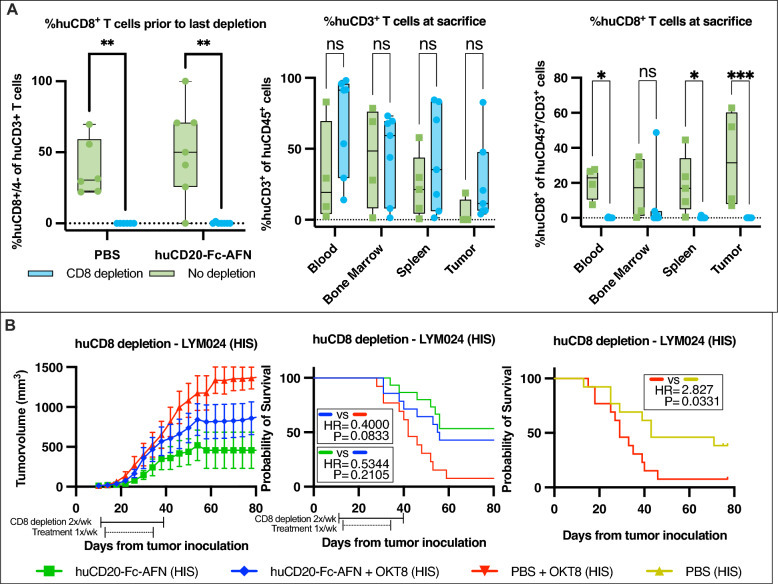
The impact of huCD8 + T cell depletion on the efficacy of huCD20-Fc-AFN. **A** Demonstration of huCD8^+^ T cell depletion by flowcytometry. Depletion was performed by OKT8 administration IP 2x/week (week 1: 200 μg/week 2–3-4: 100 μg) starting 2 days prior to start of treatments. Complete huCD8^+^ T cell depletion in the blood was confirmed prior to last depletion. Demonstration of near complete huCD8^+^ T cell depletion in blood, bone marrow, spleen, and PDX tumor up to 22 from last OKT-8 administration in combination with PBS or huCD20-Fc-AFN. No significant differences in total huCD3^+^ T cell counts were observed, indicating the selective huCD8 depletion by OKT-8 administration. **B** Tumor volumes and Kaplan Meier survival curves from tumor inoculation in HIS NSG (n = 13–14/group). Administration of huCD20-Fc-AFN 40μg 1x/week for 4 weeks from D13. PDX characteristics can be found in Table 2 and Table 1 for specific experimental conditions. *HIS*: *human immune system; HR*: *Hazard Ratio; PBS*: *Phosphate-buffered saline; AFN: AcTaferon*

The most powerful effectors in the anticancer immune response are presumed to be CD8^+^ cytotoxic T cells [51]. Previously, the presence of muCD8^+^ T cells was shown to be critical for the antitumoral efficacy of huCD20-mAFN in B16-huCD20^+^ tumors in C57BL/6J mice [15]. Therefore, we depleted huCD8^+^ cells by twice weekly IP administration of OKT8, starting 2 days prior to treatments with huCD20-Fc-AFN 40 μg 1x/week IV. Complete depletion of circulating huCD8^+^ T cells was confirmed by flow cytometry (Fig. 12A). Additionally, huCD8^+^ T cells were absent or decreased in the tumor, bone marrow and spleen for up to 22 days after last depletion (Fig. 12A). The rate of tumor growth was significantly increased upon huCD8^+^ T cell depletion in untreated tumor bearing HIS mice (mOS 20 to 43 days; HR = 2.8; p = 0.03) (Fig. 12B) confirming that huCD8^+^ T cells play an important role in tumor control in humanized mice models. Although huCD8^+^ T cell depletion also negatively impacted survival of huCD20-Fc-AFN treated mice (mOS “not reached” to 42.5 days; HR = 0.53; p = 0.21), a significant antitumoral effect was still observed despite huCD8^+^ T cell depletion (mOS 42.5 to 29 days; HR = 0.40; p = 0.08) (Fig. 12B).

### Comparison with rituximab

**Fig. 13:**
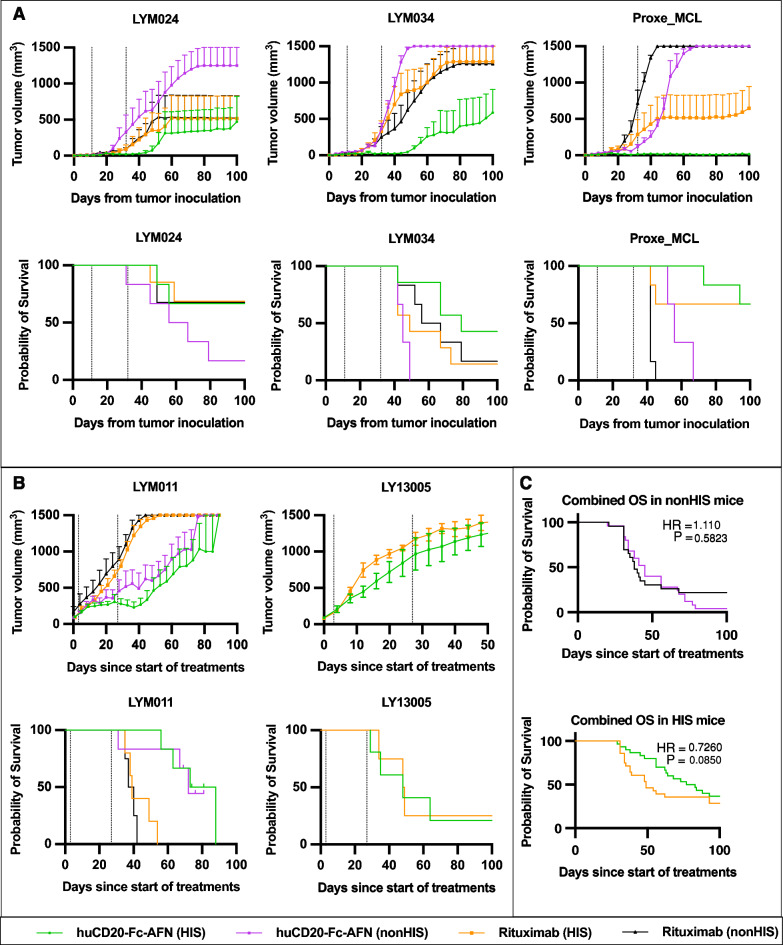
Comparison of huCD20-Fc-AFN to equimolar rituximab in 5 different humanized PDX lymphoma models. **A** Tumor volumes and survival from tumor inoculation. Administration of huCD20-Fc-AFN 40 μg or rituximab 63 μg (= equimolar) 1x/week for 4 weeks from D11. HIS and nonHIS NSG (n = 7/group). Dotted lines indicate the first and last treatment. **B** Tumor volumes and survival from start of treatment. Administration of huCD20-Fc-AFN 40 μg or rituximab 63 μg (= equimolar) 2x/week for 4 weeks, started on an individual basis when tumor volume reached > 75 mm^3^. Pretreatment with IP 30 μg huFlt3L 2 × 4 days, starting 2 days prior. For LY13005 only HIS NOG mice used (n = 5/group), for LYM011 HIS and nonHIS NSG mice (n = 5–7/group). Dotted lines indicate the first and last treatment. **C** Kaplan Meier survival curve of combined data from experiments in panel A + B. Survival from start of treatment for n = 23 vs 25 and n = 28 vs 30 in rituximab vs huCD20-Fc-AFN in nonHIS and HIS mice, respectively. A trend for improved overall survival was seen HIS mice only. PDX characteristics can be found in Table 2 and Table 1 for specific experimental conditions.* OS*: *overall survival; HR*: *Hazard ratio; HIS*: *human immune system; AFN*: *AcTaferon*

Finally, we compared the potency of huCD20-Fc-AFN with rituximab. Clinically, rituximab is dosed at 375mg/m^2^ IV, which preclinically translates to 125 mg/kg, or 2500 μg for a 20 gr mouse [52]. Equimolarly dosed, huCD20-Fc-AFN was expected to also induce effects on untargeted cells (Fig. 5). Therefore, we evaluated rituximab at 63 μg, equimolar to our standard dose of 40 μg huCD20-Fc-AFN. Figure 13A, B demonstrate the efficacy of huCD20-Fc-AFN compared to rituximab in the 5 humanized aggressive B-NHL PDX models described above. When combined, survival curves overlap in nonHIS mice (mOS 38 to 45; HR = 1.1; p = 0.58) but diverge, without reaching statistical significance, in HIS mice (mOS 49 to 80 days; HR = 0.73; p = 0.09) (Fig. 13C).

## Discussion

In this study we extended the reported potential of CD20-targeted attenuated IFNα2 in murine and cancer cell line models, to humanized PDX models as a novel approach to treat lymphoma. The high targeting efficacy, combined with half-life extension with an ‘effector-silent’ IgG1 Fc, could allow for non-toxic delivery of IFNα2 to tumors. Our results demonstrate that huCD20-Fc-AFN had a clear effect on the tumor cells directly, but an additional effect via the human immune system was present and seemed essential to obtain long-term remissions. Although our humanized PDX models attempted to mimic the clinical situation as closely as possible and included effector arms of the human immune system, they pose some known limitations. Firstly, the evaluated PDX models were all aggressive B-cell lymphomas, limiting our knowledge in indolent lymphoma subtypes. Prior rituximab exposure in 4/5 PDX models might imply an inherent resistance to rituximab. Indeed, no cytolytic effect of rituximab was observed in vitro. Nonetheless, tumors expressed huCD20, and were susceptible to huCD20-Fc-AFN treatments in vivo. Secondly, although PDX models are the closest approximation to tumors in patients, with concordance on flow cytometry and IHC, an inherent clonal selection, loss of tumor heterogeneity and microenvironment is inevitable [25, 26, 53, 54]. By limiting passages, we have tried to reduce this as much as possible. Thirdly, as recently reviewed, the humanized immune system in huNSG mice is not perfect [23]. Most human immune cell subsets can be identified; however, they are not all equally functional, such as NK cells that need huIL-15 for their maturation [55]. This lack of fully functional NK cells might explain the hampered antitumoral efficacy of rituximab [55]. The recently developed humanized NSG-Tg(Hu-IL15) model partly overcomes these limitations, and could be used to confirm the current hypothesis [56]. The antitumoral effect of huCD20-Fc-AFN was independent of additional human cDC1 expansion by exogenous huFlt3L administration and persisted despite huCD8^+^ T cell depletion. The lack of effect of cDC1 stimulation with huFtl3L is puzzling, given their central role as orchestrators of the antitumoral immune response, specifically upon IFN I stimulation [48, 57]. While many human immune cell subtypes, such as dendritic cells, are better developed in models constitutively expressing human cytokines (such as the NSG-SGM3 or NSG-Flt3L strain), it should be noted that, even without exogenous huFlt3L administration, human cDC1 are already present and functional in huNSG mice, be it in reduced numbers [45–47, 58]. Conversely, T cell priming by cDC1s predominantly occurs in tumor-draining lymph nodes, which are poorly developed in NSG mice [48, 59–61]. On the other hand, AFN targeting to cDC1 did result in antitumoral effects in HIS NSG mice [17, 20]. Consequently, our findings potentially underestimate the impact of cDC1s on huCD20-Fc-AFN efficacy in patients. Currently, we cannot explain the exact reasons why huCD20-Fc-AFN efficacy is improved upon humanization, but huCD8^+^ T cells presumably play an important effector function. Our findings demonstrated that huIFNα2, and more specifically huCD20-Fc-AFN, was not directly cytotoxic, but rather provided an in vitro survival advantage for PDX lymphoma cells. These paradoxical in vitro anti-apoptotic effects of IFNα2 on B and B-NHL cells were previously described, and are presumed to be related to reduced B cell receptor (BCR) mediated apoptosis and upregulation of the anti-apoptotic proteins Bcl-2 and Bcl-xL [62–66]. Hence, one possible explanation could be the emerging role of antigen presentation by the lymphoma itself or the (also) targeted B cells, instead of the cDC1s [66–69].

Historically, IFNα2, a highly pleiotropic cytokine, was one of the first types of immunotherapies. However, its unselective nature led to severe and potentially life-threatening toxicities such as cytopenias, hepatotoxicity, cognitive dysfunction, neurologic toxicity, debilitating depression, and even suicidal ideations. Consequently, drastic dose limitations led to reduced efficacy, as the best responses to IFNα2 were generally achieved with the highest doses [5]. The increased targeting efficacy of up to 1.000-fold due to the A-Kine principle might allow for a rebirth of IFNα2-based strategies. Due to its attenuated IFNα2-warhead, huCD20-Fc-AFN is expected to be largely inactive while traveling through the body, consequently inducing much lower systemic “background” toxicity. Additionally, besides displaying an exceptional therapeutic index, a superior pharmacokinetic behavior is expected, by limiting the so-called “sink” problem, whereby a significant portion of the administered cytokine is bound and/or taken up by the untargeted cells [16]. These properties are, at least in part, due to the 200-fold reduced affinity for the IFNAR2 receptor induced by the AFN mutation [39]. No significant in vivo toxicities related to the administration of huCD20-Fc-AFN could be observed, except for on-target depletion of circulating human B cells in humanized mice. The latter presumably due to redistribution since it was rapid, and reversible, while no in vitro cytotoxicity could be observed. However, the intrinsic species-specificity of IFN I/AFN, and lack of huCD20 expression, respectively, hamper the evaluation of most on- and off-target adverse effects on murine cells using the humanized constructs [70]. Nevertheless, the non-toxic nature of murine AFN constructs has been established previously [15, 17, 19, 20]. In conclusion, we have demonstrated in 5 different, clinically relevant, humanized, B-NHL PDX models that huCD20-Fc-AFN might provide a novel therapeutic strategy for huCD20-expressing aggressive B-NHLs. Finally, since antitumoral efficacy of huCD20-Fc-AFN increased with humanization, we hypothesize that even better responses could be expected in more immunocompetent patients.

## Data Availability

All data supporting the findings of this study are available within the paper, RNA-Seq data for DFBL-98848-V3-mCLP (Proxe_MCL) is available at https://www.ncbi.nlm.nih.gov/sra/SAMN06621890, any additional data are available from the corresponding author upon reasonable request.
